# 

**Published:** 1913-11-22

**Authors:** 


### CONTENTS.

| | PAGE |
|---|---|
| **National Insurance. The Approved Societies and the Hospitals** ... ... ... ... | 183 |
| **Progress under the Mental Deficiency Act** ... | 184 |
| **Hospital and Institutional News,** including— ... | 185 |
| New Methods in Mental Hospitals—The New Tranmere Infirmary—The Chiropody Hospital—A Tuberculosis Dispensary for Shoreditch—The Home Office and Dr. Dimock—Sir George Newman on the Open-air School—The Future of Cardiff Medical School—Wooden Huts for Consumptives. | |
| **Errors in Clinical Pathology** ... ... ... | 191 |
| By V. H. WYATT WINGRAVE, M.D. | |
| **The Evolution of the Isolation Hospital** ... | 193 |
| By the late Dr. H. FRANKLIN PARSONS. | |
| **The School Doctor's Difficulties:** ... ... | 195 |
| The Special Examination of Mental Defectives. | |
| **Collective Research for General Practitioners** | 196 |
| **Reports on Essex County Hospital and the Braintree and Bocking Cottage Hospital** ... | 197 |
| By Sir HENRY BURDETT, K.C.B., K.C.V.O. | |
| **The Heel of Achilles in Hospital Diet** ... ... | 199 |
| **A Medical Woman to Her Sex** ... ... ... | 201 |
| **Dr. W. J. C. Keats on Practical Points in Infirmary Work** ... ... ... ... ... | 202 |
| **The Provision of a Punishment Book at Camberwell Infirmary** ... ... ... ... | 203 |
| **Electric Motors as Food Carriers** ... ... | 204 |
| **The Editor's Letter-Box** ... ... ... ... | 205 |
| The London Nerve Clinic—Children in Workhouses—Marriage and Malthus | |
| **With the Wounded in Bulgaria** ... ... ... | 206 |
| **The Non-Panel Movement** ... ... ... ... | 208 |
| The Capitation Grant for Unallotted Persons. | |
| **The Institutional Worker** ... ... (Suppl.) | 1 |
| Electric Time Service for Hospitals. | |
| **Our Bureau of Information** ... ... ,, | 2 |
| **Buildings Contemplated and in Progress** ,, | 3 |
| **The Book Market** ... ... ... ,, | 3 |
| **Institutional Needs** ... ... ... ,, | 3 |

